# *QuickStats:* Percentage* of Adults Aged ≥65 Years Who Saw Selected Types of Health Professionals^^†^^ in the Past 12 Months, by Diagnosed Diabetes Status^^§^^ — National Health Interview Survey, 2015

**DOI:** 10.15585/mmwr.mm6619a10

**Published:** 2017-05-19

**Figure:**
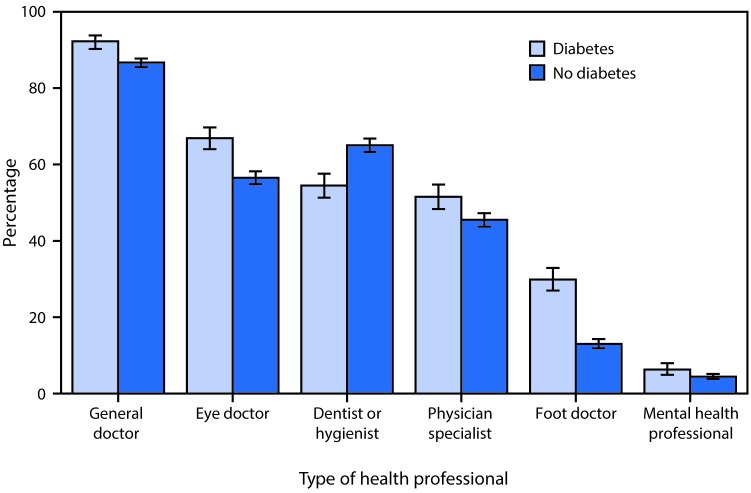
In 2015, adults aged ≥65 years with diagnosed diabetes were more likely than adults without diagnosed diabetes to report seeing general doctors (92.3% compared with 86.7%); eye doctors (66.9% compared with 56.6%); physician specialists (51.5% compared with 45.5%); foot doctors (29.9% compared with 13.0%) and mental health professionals (6.3% compared with 4.5%) in the past 12 months. Those with diabetes were less likely than those without diabetes to report seeing a dentist or dental hygienist in the past 12 months (54.5% compared with 65.0%).

**Source:** National Health Interview Survey, 2015 data. https://www.cdc.gov/nchs/nhis.htm.

